# 3D Printing of Solar Crystallizer with Polylactic Acid/Carbon Composites for Zero Liquid Discharge of High-Salinity Brine

**DOI:** 10.3390/polym15071656

**Published:** 2023-03-27

**Authors:** Qing Yin, Fangong Kong, Shoujuan Wang, Jinbao Du, Ling Pan, Yubo Tao, Peng Li

**Affiliations:** 1 State Key Laboratory of Biobased Material and Green Papermaking, Qilu University of Technology, Shandong Academy of Sciences, Jinan 250353, China; qluyinqing@163.com (Q.Y.); kfgwsj1566@163.com (F.K.); nancy5921@163.com (S.W.); zyq19980609@163.com (J.D.); 2 College of Material Science and Engineering, Northeast Forestry University, Harbin 150040, China; panling@nefu.edu.cn

**Keywords:** high-salinity brine treatment, solar crystallizer, 3D printing, salt collection, zero liquid discharge

## Abstract

Zero liquid discharge (ZLD) is a technique for treating high-salinity brine to obtain freshwater and/or salt using a solar interface evaporator. However, salt accumulation on the surface of the evaporator is a big challenge to maintaining stable water evaporation. In this study, a simple and easy-to-manufacture evaporator, also called a crystallizer, was designed and fabricated by 3D printing. The photothermal layer printed with polylactic acid/carbon composites had acceptable light absorption (93%) within the wavelength zone of 250 nm–2500 nm. The micron-sized voids formed during 3D printing provided abundant water transportation channels inside the crystallizer. After surface hydrophilic modification, the crystallizer had an ultra-hydrophilic channel structure and gravity-assisted salt recovery function. The results revealed that the angles between the photothermal layers affected the efficacy of solar evaporation and the yield of solid salt. The crystallizer with the angle of 90° between two photothermal layers could collect more solid salt than the three other designs with angles of 30°, 60°, and 120°, respectively. The crystallizer has high evaporation and salt crystallization efficiency in a high-salinity brine environment, which is expected to have application potentials in the zero liquid discharge of wastewater and valuable salt recovery.

## 1. Introduction

According to the United Nations Environment Programme, more than half of the world population will face freshwater shortage by 2025, and water scarcity and water security should be taken seriously [1,2]. In order to alleviate the shortage of freshwater resources, many countries have attached great importance to desalinations [3,4]. However, traditional desalination plants could produce a large amount of high-salinity brine in the desalination process [5,6]. This results in a large amount of untreated brine discharged directly into surface water, deep-well evaporation ponds, or the ocean, which causes water pollution and land salinization [7,8]. Therefore, zero liquid discharge (ZLD) technology was developed, which is a circular economy-based method for treating high-salinity brine, involving brine concentration and brine crystallization [9,10]. Using the ZLD technique, the dissolved minerals in the brine can be concentrated into solids for collection [11,12]. Nevertheless, traditional ZLD has shortcomings, such as low treatment efficiency, energy consumption, and high cost [13]. Reverse osmosis (RO) membrane filtration is a crucial component of traditional ZLD. The RO membrane pores could be contaminated by fouling after long periods of operation. The fouling comes from the accumulation of bacteria, sludge, metal ions, and other suspended matter. This results in a significant decline in membrane filtration performance. Therefore, the brine requires pretreatment before passing through the RO membrane for desalination [14].

In recent years, the solar-driven interfacial evaporator has been widely studied in the desalination research due to its low cost, effective use of clean energy, no carbon emission, suitability for high-salinity brine, and other benefits [15,16,17]. At present, the evaporator is mainly used for the treatment of low-salinity brine. By designing the photothermal conversion materials and structure of the evaporator, the salt inside the evaporator can quickly diffuse into water bodies [18,19]. Such an evaporator can maintain water evaporation without being affected by salt crystallization [20,21]. Although the aforementioned studies can prevent the degradation of evaporator performance caused by salt accumulation, the direct diffusion of salt will cause the salinity increase in the original water body, resulting in secondary contamination. As we know, the evaporator is also suitable for salt recovery from high-salinity brine. However, it is very easy for salt to crystallize on the evaporator surface and within the water channel while treating high-salinity brine. The efficiency of photothermal conversion and the water transport performance of evaporators will decrease due to salt accumulation [22,23]. Therefore, enabling an evaporator to have durable water evaporation performance is key to research during salt recovery.

**Figure 1:**
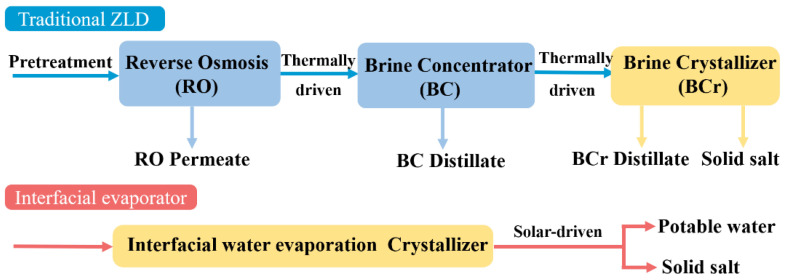
A schematic diagram of traditional ZLD and interfacial evaporator. (Reprinted with permission from Ref. [12]. 2022, Elsevier).

Some innovative interface evaporators have been designed to achieve efficient water evaporation while also enabling solid salt collection. Those ideas also realized ZLD goals of pollution-free, zero energy consumption for high-salinity brine treatment. As shown in Figure 1, the interface evaporator is able to simplify the traditional ZLD treatment process by replacing the expensive, contamination-prone RO membrane.

The research of interface evaporators focuses on material and structure design. In terms of evaporator material research, the surface of the evaporator is commonly hydrophobically modified to avoid salt crystallization, and the other part remains hydrophilic for water transmission. Gu et al. [24] modified hydrophobic carbon felt to make it hydrophilic through acid treatment, and then coated the top surface with hydrophobic poly(1,1-difluoroethylene) (PVDF). After the treatment, salt could only crystallize on the edge of evaporator, without salt accumulation on the top surface. Similarly, Dong et al. [25] first prepared hydrophilic cellulose acetate film by electrospinning. Then, through hydrophobic modification of the top surface with polydimethylsiloxane (PDMS) coating, they ensured that salt could only crystallize in the hydrophilic part of the evaporator.

In terms of evaporator structure design, some studies have realized selective salt crystallization at the edge of the evaporator by designing the evaporator’s water transport channel. Li et al. [26] prepared a water transport structure with a different bending degree using polyvinyl acetate (PVA) hydrogel. Salt could crystallize along a designated water flow direction. Bionic structural design is another route to achieve selective salt crystallization. Being inspired by the salt dilution and salt secretion mechanism of halophyte, Zhang et al. [27] prepared an evaporator capable of surface salt secretion and edge salt dilution using glass fiber felt and Polypyrrole (PPy). Sun et al. [28] 3D-printed conical PLA structures with concave grooves by mimicking mussel seashells and then coated the top layer of the evaporator with PPy to realize freshwater collection.

In the above research, although the constructed evaporators achieved the ZLD treatment goal of zero energy consumption, the coating materials or the preparation process used are costly. In addition, when the water delivery channel in the evaporator is nanoscale, Mg^2+^ and Ca^2+^ will block the channel during the desalination of high-salinity brine, which requires the addition of salt crystallization inhibitors to pretreat high-salinity brine. This also increases the cost of desalination.

With the aim of free control of the outer shape and inner water transport channel of the evaporator, seawater evaporation crystallizers were constructed by 3D-printing technology in this study. The crystallizer structure consists of two parts: (1) the top photothermal conversion layer, which seems like a sloping roof; and (2) the bottom cube for water transfer. In addition, the supporting buoyancy layer structure was designed to provide buoyancy for the crystallizer body and the solid salt collected. The photothermal conversion part was printed with Polylactic acid (PLA)/bio carbon composite filaments. The water transfer cube was printed with pure PLA filaments. Due to the hydrophobicity of PLA, the 3D-printed parts were subjected to hydrophilic modification. Considering that the slope of the photothermal conversion layer is the crucial factor that determines the effective photothermal conversion area, four types of crystallizers were constructed, with the photothermal layer containing angles of 30°, 60°, 90°, and 120°, respectively. To evaluate the salt crystallization performance of the crystallizers, the salt crystallization process was run under an extreme salt concentration environment (20 wt%). The experimental results showed that the micron-sized water transport channel and superhydrophilicity of the crystallizer produced continuous water evaporation and optimal salt crystallization performance.

## 2. Materials and Methods

### 2.1. Materials

Sodium chloride (NaCl, AR) and Tannic acid (TA, AR) were supplied by Macklin Biochemical Co., Ltd. (Shanghai, China). Polyvinylpyrrolidone (PVP, Mw~8000) was supplied by Aladdin Biochemical Technology Co., Ltd. (Shanghai, China). Iron(Ⅲ) chloride (FeCl_3_, AR) was supplied by Damao Chemical Co., Ltd. (Tianjin, China). PLA (4032D) was supplied by Nature Works LLC (Minneapolis, MN, USA). Hazelnut shells were from Benxi Agricultural Development Co., LTD. (Benxi, China).

The PLA-C filament for 3D printing was prepared by melt extrusion as follows: 98 g of PLA and 2 g of the dried hazelnut shell carbon. The preparation process of hazelnut shell carbon was as follows: in the first step, hazelnut shells were pulverized into particles. Then, hazelnut shell particles were pyrolyzed for 2 h at 700 °C under an N_2_ atmosphere in a tube furnace. The heating rate in the furnace was 5 °C per minute (OTF-1200X, KEJING, Hefei, China). Finally, further grinding was run in a ball mill machine (QM 0.4 L, Chishun LLC, Nanjing, China) and then the ground carbon powder was passed through a 120-mesh sieve and collected. The PLA was fused and blended with hazelnut shell carbon in a double roll mixer (BP-8175-A, Baopin precise instrument, Dongguan, China). The blending procedure was run at 180 °C, with the rollers’ rotation speed at 1.5 r min^−1^. Then, the blends were smashed into particles smaller than 2 mm by a disintegrator (FW135, Taisite instruments, Tianjin, China). Finally, a single-screw plastic extruder was used to extrude PLA-C filament (C2 model, Wellzoom LLC, Shenzhen, China). The extrusion temperature was in the range of 178–183 °C. Pure PLA filament was extruded under the same conditions.

### 2.2. Design and 3D Printing of the Crystallizer Structure

**Figure 2:**
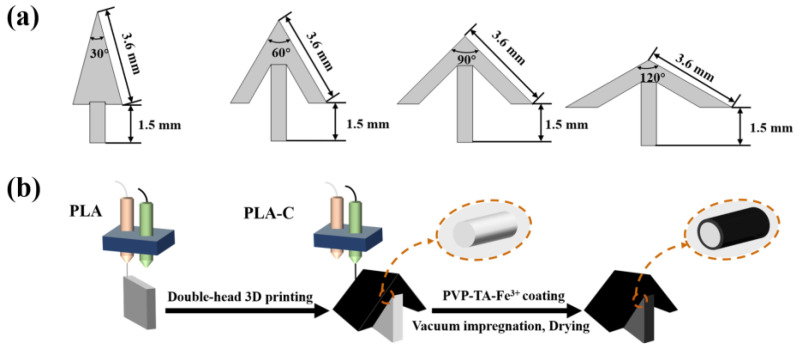
(**a**) numerical design of angle between photothermal layer of the crystallizer; (**b**) schematic diagram of crystallizer preparation process.

The model of crystallizer was designed using AutoCAD software (2022 Student edition, Autodesk software, San Rafael, CA, USA). The T-shaped crystallizer consists of two parts: the photothermal layers and the water transport layer. The detailed crystallizer design is presented in Figure 2a and the CAD drawing is provided as a supplementary document (Figure S1). The photothermal layer of the crystallizer has two rectangular water evaporation surfaces of 36 mm × 40 mm with a thickness of 6 mm. The angles between two photothermal layers were designed as 30°, 60°, 90°, and 120°, respectively. The horizontal water transport layer has a cross-section of 6 mm × 40 mm. The water transport layer has the same volume in contact with brine and a height of 15 mm. The Cura software (Cura 15.04, Ultimaker, Waltham, MA, USA) was used to slice the crystallizer model. The model slice program was set as without the exterior of the model and an infill density of 60%. An FFF (Fused Filament Fabrication) double-head printer (Inker 334, Wuhan Allcct Technology Co., Ltd., Wuhan, China) was used to construct the crystallizer. As shown in Figure 2b, the photothermal layers and the water transport layer were printed using PLA-C and PLA filament, respectively. The printing parameters were set as: a nozzle temperature of 200 °C, a hot bed temperature of 60 °C, a printing speed of 50 mm s^−1^, a printing thickness of 0.2 mm, and a nozzle diameter of 400 μm, respectively.

### 2.3. Surface Hydrophilic Modification of Crystallizer

PVP-TA-Fe^3+^ coating was synthesized to improve the hydrophilicity of the crystallizer. The preparation steps of PVP-TA-Fe^3+^ coating are as follows: PVP (0.32 g) and TA (0.32 g) were mixed in 100 mL deionized water by magnetic stirring at room temperature for 5 min. Then, FeCl_3_ (0.69 g) was added into the PVP-TA solution by magnetic stirring. The 3D-printed structure was immersed into the PVP-TA-Fe^3+^ solution and then placed in a vacuum oven for vacuum impregnation. After 12 h impregnation, the structure was dried at 50 ± 2 °C for 6 h. Eventually, the dried structure was rinsed with distilled water under ultrasound treatment for 2 h to remove the unattached PVP-TA-Fe^3+^ coating.

### 2.4. Design and 3D Printing of the Buoyant Structure

**Figure 3:**
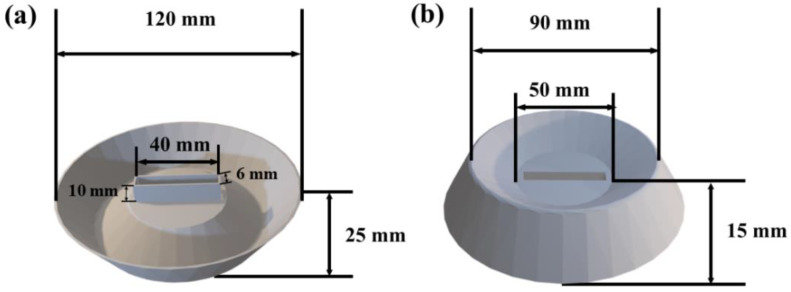
Buoyancy layer model: (**a**) front view; (**b**) back view.

The buoyancy layer can ensure that the crystallizer system floats on the brine and reduces the heat transfer between the crystallizer and brine. In this work, the buoyancy layer provided a buoyancy force greater than the total weight of the crystallizer itself, its water content, and salt crystals. The design of buoyancy layer is presented in Figure 3 and the CAD model is provided as a supplementary document (Figure S2). According to Archimedes’ theorem, the mass of the water body occupied by the buoyancy layer is equal to the buoyancy exerted on it. The following formula (1) is used to calculate the buoyancy provided by the buoyant structure [29].

F_buo_ = ρ × g × V_disp_ (1)

where ρ is brine density, 1035 kg × m^−3^, g is radio of gravity to mass, 9.8 N × kg^−1^, V_disp_ is volume of displacement, m^3^.

### 2.5. Characterization

The microstructure of the crystallizer was observed by scanning electron microscopy at 5 kV (SEM, JSM-7401F, Hitachi, Tokyo, Japan). The contact angle was recorded to compare the wettability of the crystallizer surface (the optical contact angle, Dataphysics OCA20, Filderstadt, Germany). The tensile specimens were designed according to ASTM D638 Type IV [30]. All samples were 3D-printed as described in Section 2.2. Tensile tests were conducted by a universal tensile machine (TSE104B, Wance, Shenzhen, China) with the tensile rate of 5 mm/min^−1^. The reflectance (R%) and transmittance (T%) of the crystallizer at 250 nm–2500 nm were tested by Ultraviolet-visible-near-infrared spectrophotometer (UV-VIS-NIR, Cary 7000, Santa Clara, CA, USA). The light absorbance (A%) of the crystallizer was calculated by the equation: A% = 1 − (R% + T%).

### 2.6. Salt Crystallization Test

The photothermal conversion and crystallization capabilities of the crystallizer were evaluated under a customized solar simulation test system (Xenon light source, CME-SL500, Micro energy, Beijing, China). A solar power meter (TES132, ShenZhen, China) was used to maintain the surface light intensity of the crystallizer at 1000 W m^−2^. An electronic balance equipped with a computer was adopted to record the water mass change continuously. The accuracy of the balance was 0.0001 g (BSA224S, Sartorius, Gottingen, Germany). The surface temperature of the crystallizer was monitored with an infrared thermal camera (FLIR E8, Wilsonville, OR, USA). During the water evaporation test, the crystallizer was embedded in the expandable polyethylene foam (EPF) to isolate the natural evaporation of bulk water. This also ensured that the photothermal layer of the crystallizer can convert light to heat and promote the evaporation of brine to generate steam at the interface. To test the water evaporation of the crystallizer, a 50 mL quartz beaker was used to hold the crystallizer, high-salinity brine, and EPF. To test the continuous salt crystallization of the crystallizer, a glass flume was used to hold the crystallizer, high-salinity brine, and buoyant structure. The test was conducted at an ambient temperature (20 ± 2 °C) and relative humidity (50 ± 5%).

### 2.7. COMSOL Simulation Test

The mass transfer processes of water and salt include advection, diffusion, and evaporation. The laminar flow and dilute material transfer were used for evaluating the ion diffusion in the crystallizer. The distribution of ion concentration was studied by solving the following equation:

ρ × əu/ət + ρ × (u × ∇u) × u = ∇[−pI + K] + F + ρg(2)

ρ × ∇u = 0(3)

əc/ət + ∇J + u × ∇c = R(4)

J = −D × ∇c(5)

where u is the velocity field, set as 4 × 10^−7^ m × s^−1^; I is the constitutive relation coefficient; K is the viscosity, set as 8.9 × 10^−4^ Pa·s; ρ is the brine density, set as 1 035 kg × m^−3^; F is the surface tension at the interface between air and liquid, set as 0. R is the reaction on the ion surface, set as 0; c is the ion concentration, set as 3589 mol × L^−1^, corresponding to 20 wt% brine; t is the time, and J is the concentration gradient.

## 3. Results and Discussion

### 3.1. Preparation and Surface Characterization of Crystallizer

**Figure 4:**
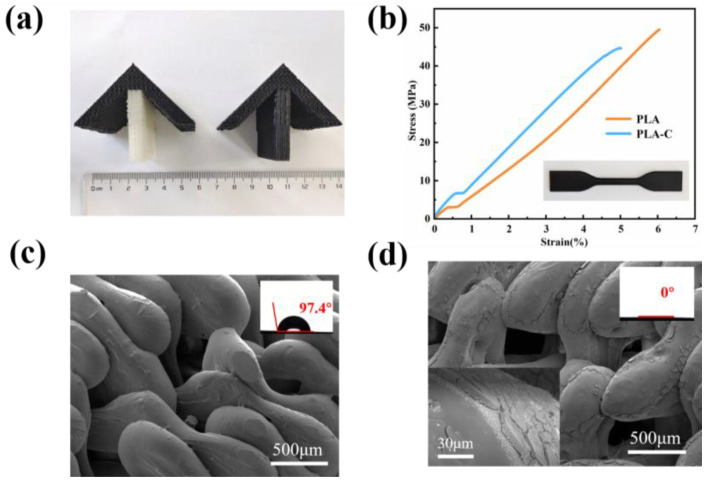
(**a**) digital photograph of the 3D-printed crystallizer structure without and with coating; (**b**) tensile properties of the standard tensile specimen; (**c**) Microstructure and wettability of crystallizer without PVP-TA-Fe^3+^ coating; (**d**) Microstructure and wettability of crystallizer with PVP-TA-Fe^3+^ coating.

Bio-based carbon composites are ideal photothermal materials due to their advantages such as being robust and inexpensive, and their capability to absorb full-spectrum solar wavelengths [31,32,33]. In this study, 3D printing was combined with bio-based photothermal materials to construct controllable crystallizer structure. As shown in Figure 4a, the crystallizer structure built by 3D printing consists of the black part printed with PLA-C filament and the white part printed with PLA filament. PLA-C and PLA filaments were used to construct the photothermal layer and water transfer layer of the crystallizer, respectively. After being printed, these two parts were integrated together to form the main structure of the crystallizer. Then, the crystallizer structure was treated with a PVP-TA-Fe^3+^ coating. After PVP-TA-Fe^3+^ treatment, the whole structure became significantly black. The PVP-TA-Fe^3+^ system utilized its hydrophilic as well as strong adhesive property to form a stable coating [34,35,36]. As shown in Figure 4c, the surface of micron-sized water transport channels without coating was smooth. In comparison, the surface of the structure became rough after coating (Figure 4d). The contact angle results in Figure 4c, d proved that the hydrophobic water transfer channels were modified to be ultra-hydrophilic after the coating treatment. Furthermore, the mechanical property of the crystallizer is crucial for stable operation of the evaporator system. The tensile experiments demonstrated that all specimens constructed by 3D printing had a tensile strength above 45 MPa, as shown in Figure 4b.

### 3.2. Buoyant Structure

The hollow cone-shaped buoyant layer was designed parametrically by CAD. The center of the buoyancy layer holds the crystallizer, and the concave groove of the buoyancy layer collects the dislodged salt crystals. The buoyancy layer also acts as an insulator, reducing the heat transfer between the crystallizer and the brine. According to the buoyancy formula (1) and the specific design data of the buoyancy layer, it can be calculated that the volume of brine discharged by the buoyancy layer was 158.57 cm^3^. Therefore, the buoyancy layer can enable an object weighing 168.375 g to float on the brine. In this experiment, the buoyancy layer itself weighed 21.64 g, and the weight of crystallizer containing brine was 25 ± 5 g. Therefore, the buoyancy layer can float an object weighing 120 ± 5 g on the brine, which indicates that it can provide a stable salt crystallization environment for the crystallizer.

### 3.3. Salt Crystallization Performance of the Crystallizer

**Figure 5:**
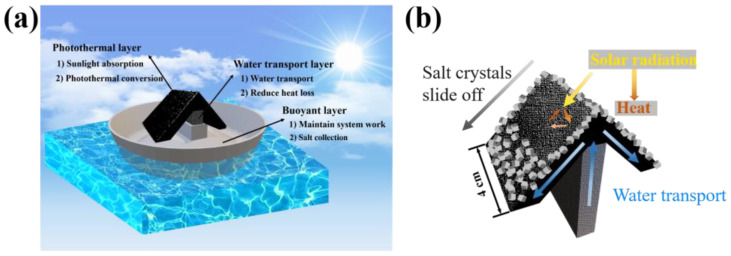
(**a**,**b**) schematic illustration of crystallizer based on 3D printing.

The hydrophilic coating could ensure efficient water delivery in the micron-sized channels of the crystallizer. However, salt crystals may accumulate on the photothermal layer during continuous water evaporation, which affects its evaporation efficiency. The schematic illustration of the assembly of crystallizer device is shown in Figure 5. The whole crystallizing system consists of the photothermal layer, water transport layer, and buoyancy layer. The function of the water transport layer is to transfer brine from the water body to the photothermal layer. The function of the photothermal layer is to absorb solar radiation and convert solar energy into heat, warming and evaporating brine to obtain salt crystals. The buoyancy layer collects the salt crystals produced during the stable operation of the entire system. By regulating the difference in surface area between the photothermal layer and the water transport layer, the salt can crystallize on the surface of the photothermal layer. Meanwhile, the angle between two photothermal layers was designed to ensure that the salt crystals can fall off on their own by gravity, which supports the stable operation of the crystallizer.

**Figure 6:**
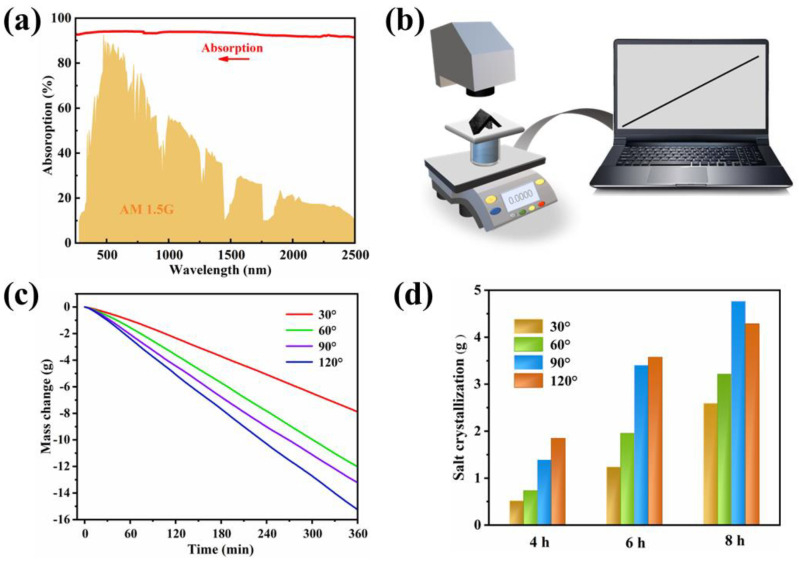
(**a**) Light absorption of crystallizer at the 250−2500 nm wavelength spectrum; (**b**) schematic of an indoor solar evaporation setup; (**c**) mass change of the brine over time at different crystallizer angles; (**d**) salt collection of different crystallizers within three durations of test.

Excellent photothermal conversion performance can promote water evaporation and salt crystallization. The solar absorption of the crystallizer at the full-spectrum wavelength is shown in Figure 6a, reaching 93%. It indicated that the photothermal layer of the crystallizer had good light-absorption capacity. As shown in Figure 6b, an indoor solar simulation device was used to evaluate the water evaporation performance. As shown in Figure 6c, the water evaporation rate of the crystallizer increased as the angle between the photothermal layers increased. This is due to the height of the crystallizer itself becoming smaller as the angle between the photothermal layers becomes larger, and then the brine was able to reach the photothermal layer more quickly. After the crystallizer had been running for a specified period, the solid salt was collected from the crystallizer surface as well as from the buoyancy layer. The solid salt collected was dried at 55 °C for 6 h to a constant weight. As shown in Figure 6c, d, the angle between the photothermal layers played a key role in the evaporating rate of the crystallizer. During a specific operation period, brine mass decreased faster as the angle of photothermal layer inclusion increased. Correspondingly, the amount of solid salt collected followed the opposite trend. However, a larger angle between the photothermal layers does not mean better salt crystallization. When the system had been operating for 8 h, the crystallizer with an inclusion angle of 120° collected less solid salt than the one with a 90° inclusion angle. This is due to the fact that the solid salt on the photothermal surface of the crystallizer with an inclusion angle of 90° was subjected to a larger gravitational component parallel to the slope than the one with a 120° inclusion angle. As the photothermal conversion process continued, the mass of salt crystals on the slope increased. When the gravitational component parallel to the slope overcomes the friction between the salt and the photothermal surface, the salt will drop off. However, at an inclusion angle of 120°, it was more difficult for solid salt falling off from the evaporation surface, resulting in more salt accumulation on the slope, which reduced its salt collecting efficacy.

**Figure 7:**
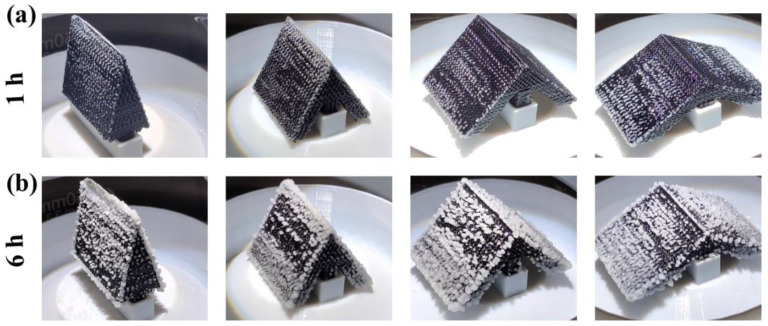
Digital photograph of salt crystallization: (**a**) 1 h operation of the crystallizer; (**b**) 6 h operation of the crystallizer.

Figure 7 demonstrated the distribution of salt crystals on the surface of the crystallizers at different angles after 1 h and 6 h of operation. After 1 h of operation, salt crystals appeared on the surface of all crystallizers. After 6 h of operation, the top edge of the crystallizer at 30° and 60° were covered with salt crystals, while the top edge of the crystallizers at 90° and 120° did not show this phenomenon. The reason for this is that the brine was not replenished after evaporation, resulting in salt crystal accumulation on the surface of the photothermal layer. The salt crystalline encrustation could reduce the photothermal efficiency of the crystallizer, which is detrimental to the long-term operation of the crystallizer. In contrast, the top edges of the crystallizers with inclusion angles of 90° and 120° showed no salt crystals wrapped around them, proving that their photothermal layer enabled rapid brine replenishment along with water evaporation. Among four types of crystallizers, the water transfer distance from the brine to the photothermal layer of the crystallizers with inclusion angles of 30° and 60° is longer, and the water transfer efficiency is lowered.

### 3.4. Simulation of Salt Ion Distribution on the Crystallizer Surface

**Figure 8:**
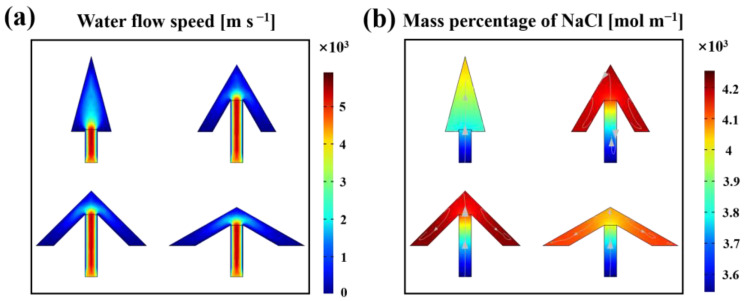
(**a**) Simulated water flow speed; (**b**) simulated salt concentration distribution(arrows is the direction of water flow).

To perceive the water transportation and the ion concentration distribution during the operation of the crystallizer, the salt ion distribution in the crystallizer was simulated. The profile structure of the crystallizers was simulated using COMSOL Multiphysics software. The results of the water transport simulation are shown in Figure 8a. It is clear that the brine was quickly transported from the water transport layer to the photothermal layer. During brine transportation, brine backflow as well as diffusion occurred simultaneously. As the inclusion angle between the photothermal layers became bigger, the diffusion effect of brine became stronger at the intersection of the photothermal layer and the water transport layer. At the same time, the backflow effect of brine became more pronounced. The results of the salt concentration distribution simulation are shown in Figure 8b. The salt concentration within the water transport layer maintained a bottom-up increase, whereas the salt concentration within the photothermal layer changed from the center to the edges. The salt concentration in the photothermal layer were higher in the crystallizers with inclusion angles of 60° and 90° than in those with 30° and 120° inclusion angles. A higher salt concentration indicated a trend towards better salt crystallization on the photothermal layer.

### 3.5. Long-Term Stability of the Crystallizer at 90°

**Figure 9:**
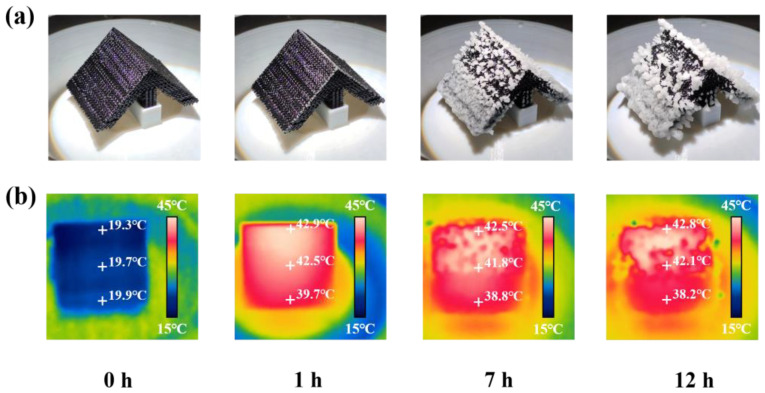
During 12 h operation of the crystallizer: (**a**) Digital photograph of salt crystallization; (**b**) infrared thermal images.

Through experiments and simulations, it can be seen that the crystallizer with an inclusion angle of 90° had higher salt crystallization efficiency. To evaluate the long-term stability of the crystallizer at 90°, a 12 h run was recorded and analyzed. As shown in Figure 9a, it is obvious that salt crystals continuously generated on the surface of the crystallizer with the increase of time. Though the salt mass rose with time, the pores in the salt crust ensured successful water transportation [37]. During the evaporation process, the salt ion concentration could keep in a dynamic equilibrium. Salt crystals would slide off the photothermal layer when the mass of the salt crystals was large enough [38]. The shedding of salt crystals ensured that the upper of the photothermal layer was not blocked by salt crystals, allowing the crystallizer to operate for a long time to produce salt crystals. Figure 9b shows that under the irradiation of one-sun illumination, the surface temperature on the upper and middle parts of the photothermal layer was maintained at 42 ± 2 °C, and the lower part of it was maintained at 38 ± 2 °C. Although salt accumulated at the lower part of the photothermal layer, which led to the temperature drop, the overall temperature of the photothermal layer still showed a high temperature. This was due to the upper and middle parts of the photothermal layer having a continuous photothermal conversion ability, which allowed the evaporation surface to transfer heat quickly and ensured the continuous salt crystallization capacity of the photothermal layer.

## 4. Conclusions

In this work, a seawater evaporation crystallizer with designated geometry and an inner channel was constructed through 3D printing. This crystallizer was capable of producing salt crystals during the treatment of high-salinity brine. The buoyancy layer in the design not only provided buoyancy but also acted as salt collector and could reduce the overall heat loss of the crystallizer. Through experiment and simulation analyses, it was determined that the salt crystallizer with an angle of 90° between the photothermal layers could maintain continuous salt crystallization and a higher efficiency of salt harvesting in a high-salinity brine environment. This crystallization system has long-term stability in the treatment of high-salinity brine and would have practical application in achieving the zero liquid discharge of high-salinity water and the recovery of valuable salts.

## Data Availability

The data presented in this study are available on request from the corresponding author.

## Supplementary Materials

The following supporting information can be downloaded at: https://www.mdpi.com/article/10.3390/polym15071656/s1, Figure S1: parameters for designing crystallizers; Figure S2: model of buoyancy layer.
