# Supplementary material for: 3D Printing of Solar Crystallizer with Polylactic Acid/Carbon Composites for Zero Liquid Discharge of High-Salinity Brine
Source: Polymers (Basel). 2023 Mar 27;15(7):1656. doi: 10.3390/polym15071656 (PMC10096562; doi:10.3390/polym15071656)
Supplement: Supplementary file 1 [file polymers-15-01656-s001.zip › polymers-2263045-supplementary.pdf]

Supplementary Materials: 3D printing of Solar Crystallizer with Polylactic Acid/Carbon Composites for Zero Liquid Discharge of High-Salinity Brine

Qing Yin [1], Fangong Kong [1], Shoujuan Wang [1], Jinbao Du [1], Ling Pan [2], Yubo Tao [1,*] and Peng Li [1,2,*]

[1] State Key Laboratory of Biobased Material and Green Papermaking, Qilu University of Technology, Shandong Academy of Sciences, Jinan 250353, China; qluyinqing@163.com (Q.Y.); kfgwsj1566@163.com (F.K.); nancy5921@163.com (S.W.); zyq19980609@163.com (J.D.)
[2] College of Material Science and Engineering, Northeast Forestry University, Harbin 150040, China; panling@nefu.edu.cn
* Correspondence: taoyubo@qlu.edu.cn (Y.T.); lipeng@qlu.edu.cn (P.L.)

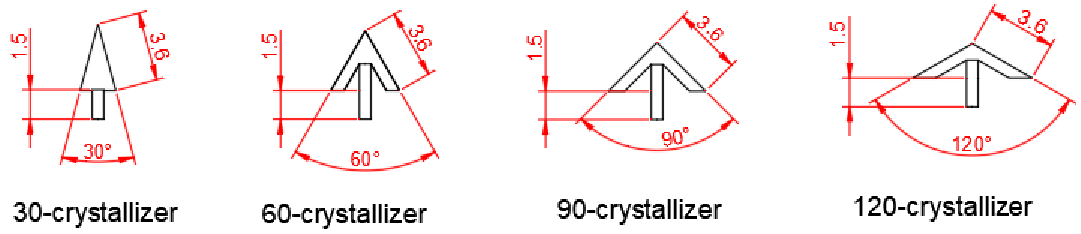

Figure S1. parameters for designing crystallizers

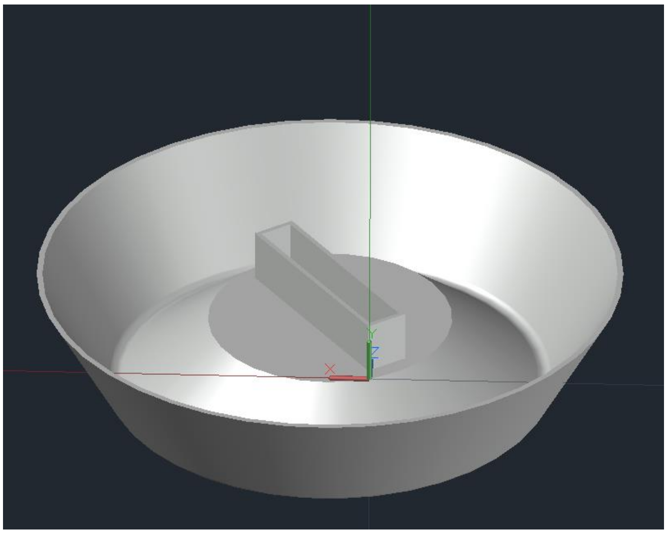

Figure S2. model of buoyancy layer
